# The elevated preoperative fasting blood glucose predicts a poor prognosis in patients with esophageal squamous cell carcinoma: The Fujian prospective investigation of cancer (FIESTA) study

**DOI:** 10.18632/oncotarget.11247

**Published:** 2016-08-12

**Authors:** Dan Hu, Feng Peng, Xiandong Lin, Gang Chen, Binying Liang, Chao Li, Hejun Zhang, Xuehong Liao, Jinxiu Lin, Xiongwei Zheng, Wenquan Niu

**Affiliations:** ^1^ Department of Pathology, Fujian Provincial Cancer Hospital, The Affiliated Hospital of Fujian Medical University, Fuzhou, Fujian, China; ^2^ Department of Cardiology, The First Affiliated Hospital of Fujian Medical University, Fuzhou, Fujian, China; ^3^ Medical-Record Department, Fujian Provincial Cancer Hospital, The Affiliated Hospital of Fujian Medical University, Fuzhou, Fujian, China; ^4^ State Key Laboratory of Medical Genomics, Rui Jin Hospital, Shanghai Jiao Tong University School of Medicine, Shanghai, China

**Keywords:** fasting blood glucose, esophageal squamous cell carcinoma, prognosis, mortality, FIESTA study

## Abstract

Diabetes as a latent risk factor for cancer has been extensively investigated, while its postoperative prognosis for esophageal cancer is rarely reported. We therefore sought to assess whether the elevated fasting blood glucose before surgery was associated with poor survival in esophageal cancer patients by eliciting a subset of data from the ongoing Fujian prospective investigation of cancer (FIESTA) study. Over 15-year follow-up, 2535 patients receiving three-field lymphadenectomy were assessable. Only patients with esophageal squamous cell carcinoma (ESCC) (n=2396) were analyzed due to the lower prevalence of the other histological types. In ESCC patients, the follow-up duration ranged from 0.5 to 180 months (median 38.2 months). The median survival time (MST) was remarkably shorter in males than in females (80.7 vs. 180+ months, Log-rank test: P<0.001). In males, the survival was worse in patients with diabetes than those without (MST: 27.9 vs. 111.1 months, Log-rank test: P<0.001). In females, the survivor was improved in patients with diabetes (MST: 71.5 months), but was still worse than patients without diabetes (MST: 180+ months, Log-rank test: P<0.001). The overall multivariate hazard ratio for per unit increment in fasting blood glucose was 1.11 (95% confidence interval or CI: 1.09-1.14, P<0.001) and 1.08 (95% CI: 1.03-1.13, P=0.002) in males and females, respectively. Further survival tree analysis consolidated the discrimination ability of fasting blood glucose for the survival of ESCC patients. Taken together, our findings convincingly demonstrated that the elevated preoperative fasting blood glucose can predict poor survival of ESCC patients, especially in males.

## INTRODUCTION

In recent two decades, esophageal cancer is soaring to epidemic proportions globally, and it ranks as the fourth most common cancer in China [1, 2]. There are two major histological types of esophageal cancer, *viz.* esophageal squamous cell carcinoma (ESCC) and esophageal adenocarcinoma (EAC). Thereof, ESCC has a high frequency of occurrence in China. Despite therapeutic advances in clinical management, the prognosis of esophageal cancer is still unsatisfactory, with the 5-year survival rate of around 20% in general populations [3, 4]. To improve the overall survival rate of esophageal cancer patients, it is clinically practicable to identify effective and easily obtained biomarkers with prognostic significance to guide treatment decisions.

A large number of case-control and cohort studies have showed that diabetes is associated with the significant risk of suffering esophageal cancer [5–8]; however, the results are often not reproducible [9–11]. According to a comprehensive meta-analysis, the diabetes-associated risk for esophageal cancer was significantly increased in males, but not in females [12]. However, data regarding the prognostic impact of blood glucose or diabetes on esophageal cancer mortality are lacking in medical literature, except for a prospective cohort study by Backemar et al, who in 609 postoperative patients from Sweden reported no strongly increased risk for esophageal cancer mortality in diabetic patients [13]. To fill this gap in knowledge and yield a more precise estimate, we sought to assess whether the elevated fasting blood glucose before surgery was associated with a poor prognosis in postoperative esophageal cancer patients by eliciting a subset of data from an ongoing Fujian prospective investigation of cancer (FIESTA) study.

## RESULTS

### Follow-up observation

In this cohort study, a total of 2535 assessable patients aged 30-88 years were analyzed. The median survival time was 44.0 months, and the 5-year survival rate was 52.2%, which was comparable with that of previous reports adopting three-field lymphadenectomy [14, 15]. According to histological types, 2396 patients had ESCC, 83 patients had EAC and 56 patients had esophageal neuroendocrine carcinomas. Due to the small number of patients with EAC and esophageal neuroendocrine carcinomas, the following analyses were restricted to ESCC patients (n=2396) only, including 1822 males and 574 females. In ESCC patients, the follow-up duration ranged from 0.5 to 180 months (median 38.2 months).

### Baseline characteristics

The gender-stratified comparisons of baseline characterizes in ESCC patients are presented in Table 1. Male patients tended to be younger and have lower body mass index, but they had higher proportions of smokers and drinkers (all P<0.001). The percentage of family cancer history and the median level of tumor size were higher in males than in females (P=0.026 and <0.001, respectively). The distributions of depth of invasion, regional LNM and TNM stage differed significantly between genders (all P<0.001). No difference was observed for fasting blood glucose (P=0.142), esophagus location (P=0.205), histological differentiation (P=0.838) and vascular cancer embolus (P=0.089). The overall survival time between never and ever smokers (MST: 92.2 vs. 63.7 months, Log-rank test: P=0.036) was marginally significant in males, whereas no significance was observed in female smokers, as well as between never and ever drinkers in both genders (Supplementary Figure S1 and Supplementary Figure S2).

**Table 1:** The comparisons of baseline characteristics between male and female patients

Characteristics	Males (n=1822)	Females (n=574)	P
Age (years)	55.98±9.81	57.93±9.41	<0.001
Body mass index (kg/m^2^)	22.14±2.90	22.83±3.26	<0.001
Ever smoking	54.13%	2.81%	<0.001
Ever drinking	25.96%	1.31%	<0.001
Family cancer history	14.70%	10.90%	0.026
Fasting blood glucose (mmol/L)	5.14 (4.60, 6.53)	5.32 (4.76, 6.62)	0.142
Esophagus location			0.205
Upper	9.85%	10.52%	
Middle	79.74%	81.75%	
Lower	10.41%	7.74%	
Histological differentiation			0.838
Well	15.26%	14.63%	
Moderate	66.74%	66.38%	
Poor	18.00%	18.99%	
Depth of invasion			<0.001
T1-T2	24.52%	40.53%	
T3-T4	75.48%	59.47%	
Regional lymph node metastasis			<0.001
N0	38.97%	49.83%	
N1	28.65%	28.05%	
N2	21.02%	16.03%	
N3	11.36%	6.10%	
Vascular cancer embolus (+)	17.12%	14.11%	0.089
TNM stage			<0.001
I	7.66%	14.41%	
II	30.71%	38.49%	
III	61.63%	47.10%	
Tumor size (cm)	4.50 (3.00, 6.00)	4.00 (2.80, 5.00)	<0.001

*Abbreviations*: TNM, tumor-node-metastasis. Data are expressed as mean ± standard deviation or median (interquartile range) or percentage. P was calculated by the t test or the Mann-Whitney U Test or the Chisq test where appropriate.

### Survival estimates: diabetes

**Figure 1:**
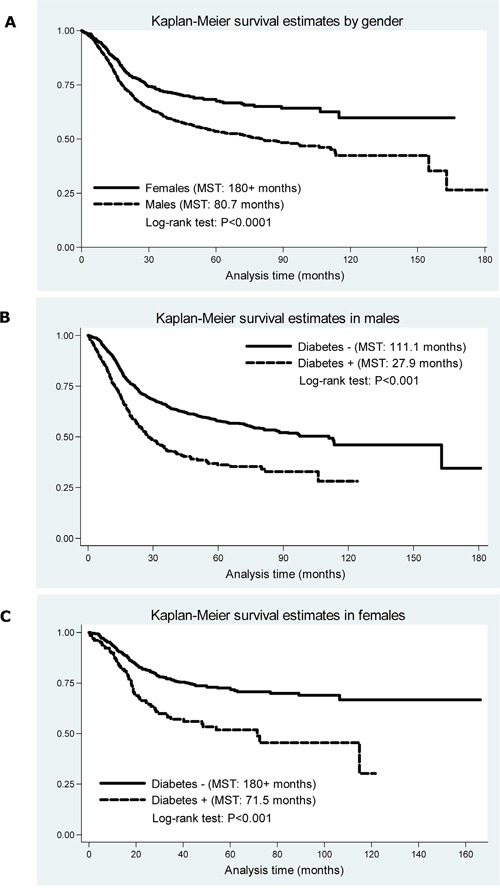
The Kaplan-Meier survival curves by gender **A.** and by diabetes in both genders **B.** and **C.** *Abbreviations*: MST, median survival time. The vertical axis represents the cumulative survival rate.

In view of the gender-specific association between diabetes and esophageal cancer risk according to a previous report [12], the overall survival comparisons of ESCC patients between genders were firstly examined (Figure 1A). The MST in males was found to be significantly shorter than that in females (80.7 vs. 180+ months, Log-rank test: P<0.001). Because of this marked difference, the following analyses were carried out by gender. Next in males, the prognosis of ESCC was worse in patients with diabetes than those without (MST: 27.9 vs. 111.1 months, Log-rank test: P<0.001) (Figure 1B). In females, the survival was improved in patients with diabetes (MST: 71.5 months), but was still worse than patients without diabetes (MST: 180+ months, Log-rank test: P<0.001) (Figure 1C).

### Survival estimates: fasting blood glucose

Overall and stratified risk predictions of fasting blood glucose for ESCC mortality before and after adjusting for age, body mass index, smoking, drinking and family cancer history are summarized in Table 2. The overall multivariate HR for per unit increment in fasting blood glucose was 1.11 (95% CI: 1.09-1.14, P<0.001) in males and 1.08 (95% CI: 1.03-1.13, P=0.002) in females. In stratified analyses, the risk prediction for per unit increment in fasting blood glucose was reinforced in male patients with TNM stage: I-II (adjusted HR=1.16, 95% CI: 1.10-1.22, P<0.001), depth of invasion: T1-T2 (adjusted HR=1.19, 95% CI: 1.11-1.27, P<0.001) and regional LNM: N0 (adjusted HR=1.18, 95% CI: 1.12-1.24, P<0.001), even after adjusting for confounding factors mentioned above. Contrastingly in females, per unit increment in fasting blood glucose was significantly associated with ESCC mortality in patients with depth of invasion: T3-T4 (adjusted HR=1.07, 95% CI: 1.02-1.13, P=0.012) and negative embolus (adjusted HR=1.08, 95% CI: 1.02-1.15, P=0.005) after adjustment.

**Table 2:** Overall and stratified predictions of preoperative fasting blood glucose (mmol/L, per unit increment) for the prognosis of ESCC patients

Group	Males		Females	
	HR, 95% CI, P	adj-HR, 95% CI, P	HR, 95% CI, P	adj-HR, 95% CI, P
Overall	1.17, 1.09-1.14, <0.001	1.11, 1.09-1.14, <0.001	1.10, 1.05-1.15, <0.001	1.08, 1.03-1.13, 0.002
Subgroups				
TNM stage: I-II	1.18, 1.12-1.24, <0.001	1.16, 1.10-1.22, <0.001	1.12, 1.014-1.24, 0.024	1.11, 1.00-1.23, 0.052
TNM stage: III-IV	1.09, 1.06-1.12, <0.001	1.09, 1.06-1.11, <0.001	1.07, 1.02-1.13, 0.008	1.05, 0.99-1.10, 0.108
Depth of invasion: T1-T2	1.19, 1.12-1.27, <0.001	1.19, 1.11-1.27, <0.001	1.07, 0.95-1.20, 0.220	1.04, 0.93-1.18, 0.482
Depth of invasion: T3-T4	1.10, 1.07-1.13, <0.001	1.09, 1.07-1.12, <0.001	1.09, 1.04-1.15, 0.001	1.07, 1.02-1.13, 0.012
Regional LNM: N0	1.19, 1.14-1.25, <0.001	1.18, 1.12-1.24, <0.001	1.11, 1.00-1.23, 0.047	1.10, 1.00-1.22, 0.061
Regional LNM: N1-N3	1.09, 1.06-1.12, <0.001	1.09, 1.06-1.11, <0.001	1.08, 1.02-1.13, 0.005	1.05, 0.99-1.11, 0.085
Negative embolus	1.12, 1.09-1.16, <0.001	1.12, 1.08-1.15, <0.001	1.10, 1.04-1.16, 0.001	1.08, 1.02-1.15, 0.005
Positive embolus	1.08, 1.04-1.12, <0.001	1.08, 1.04-1.13, <0.001	1.06, 0.97-1.15, 0.186	1.04, 0.94-1.14, 0.483
Tumor size ≤ 4.5 (M)/4.0 (F) cm	1.15, 1.11-1.18, <0.001	1.14, 1.10-1.18, <0.001	1.11, 1.05-1.17, <0.001	1.08, 1.02-1.15, 0.005
Tumor size > 4.5 (M)/4.0 (F) cm	1.09, 1.06-1.13, <0.001	1.09, 1.05-1.12, <0.001	1.08, 0.99-1.18, 0.070	1.08, 0.99-1.18, 0.089

*Abbreviations*: HR, hazard ratio; 95% CI, 95% confidence interval; TNM, tumor-node-metastasis; LNM, lymph node metastasis; M, males; F, females. *P was calculated by the Weibull proportional hazards regression model after adjusting for age, body mass index, smoking, drinking and family cancer history.

Moreover, ESCC patients were further divided into quartiles based on fasting blood glucose in males and females, respectively (Supplementary Figure S3). In both genders, patients in the highest quartile had the worst survival for ESCC. However, male patients in the second quartile and female patients in the third quartile had the best survival.

### Survival tree analysis: fasting blood glucose

**Figure 2:**
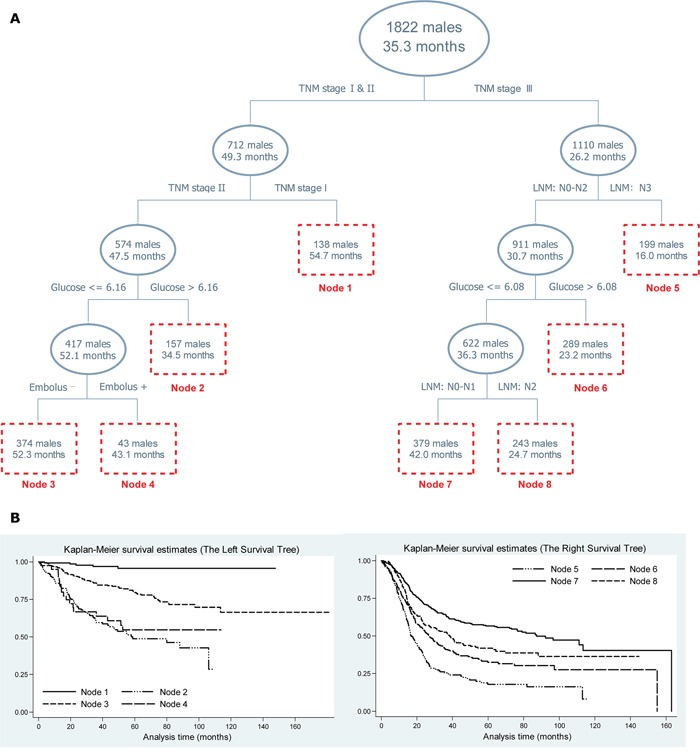
Survival tree structure **A.** and the corresponding Kaplan-Meier curves of end nodes in survival tree **B.** in males. *Abbreviations*: TNM, tumor-node-metastasis; LNM, lymph node metastasis; glucose, fasting blood glucose in mmol/L. The upper number in the box represents the number of patients, and the lower number represents the median follow-up time. The vertical axis in pane B represents the cumulative survival rate.

**Figure 3:**
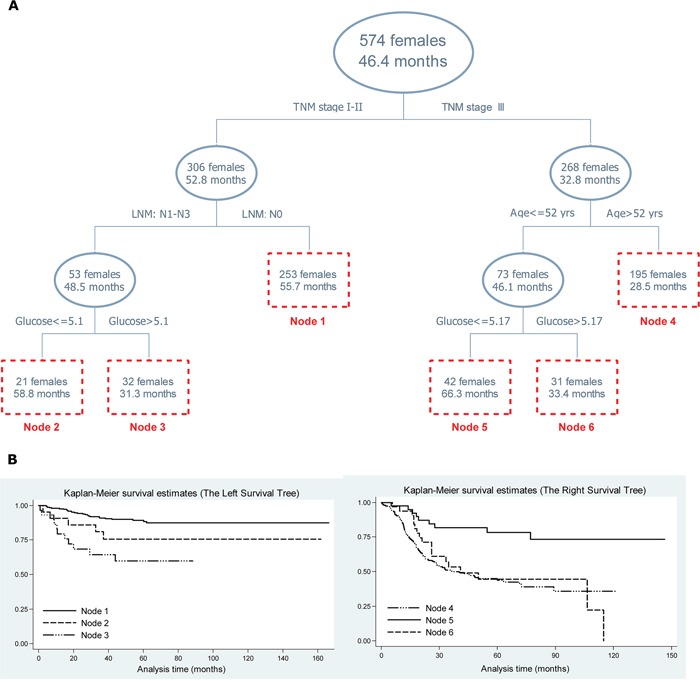
Survival tree structure **A.** and the corresponding Kaplan-Meier curves of end nodes in survival tree **B.** in females. *Abbreviations*: TNM, tumor-node-metastasis; LNM, lymph node metastasis; glucose, fasting blood glucose in mmol/L; yrs, years. The upper number in the box represents the number of patients, and the lower number represents the median follow-up time. The vertical axis in pane B represents the cumulative survival rate.

Survival tree analysis was employed to determine the optimal cutoff value of fasting blood glucose in ESCC patients, together with demographic and clinicopathologic variables under study in a gender-specific manner (Figure 2 and Figure 3). In both genders, the top splitting factor was TNM stage, followed by regional LNM and fasting blood glucose (Figure 2A and Figure 3A). Especially in males with TNM stage II, ESCC patients with fasting blood glucose ≤ 6.16 mmol/L had significantly longer median follow-up time than patients with fasting blood glucose > 6.16 mmol/L (52.1 vs. 34.5 months, Log-rank test: P<0.001), as further confirmed by the Kaplan-Meier curves (Figure 2B). In females with TNM stage: I-II and regional LNM: N1-N3, ESCC patients with fasting blood glucose ≤ 5.1 mmol/L had significantly longer median follow-up time than patients with fasting blood glucose > 5.1 mmol/L (58.8 vs. 31.3 months, Log-rank test: P<0.001), as further validated by the Kaplan-Meier curves (Figure 3B).

## DISCUSSION

In this FIESTA study, our findings convincingly demonstrated that the elevated preoperative fasting blood glucose can predict poor survival of ESCC patients, and this prediction was much better in males. More practically, the presence of diabetes in ESCC patients was associated with a worse survival experience than its absence, calling for close monitoring of blood glucose when appraising the prognosis of postoperative ESCC patients in routine clinical practice. Our survival tree analysis further consolidated the discrimination ability of fasting blood glucose for the survival of ESCC patients. To the best of our knowledge, this is to-date the largest prospective cohort study that has interrogated the postoperative prognosis of preoperative fasting blood glucose for esophageal cancer.

Diabetes as a latent predictive risk factor for a wide range of malignancies has been extensively investigated [16–18], while its prognostic impact on the survival of esophageal cancer patients is rarely seen in medical literature. One possible reason is due to the unbalanced international distributions of esophageal cancer. Unlike many Western countries where EAC dominates, ESCC is the major type in China and accounts for over 90% of esophageal cancer cases [2, 19]. As a highly aggressive malignancy, esophageal cancer is characterized by rapid growth and early metastasis. Indeed, the majority of patients with esophageal cancer have entered into an advanced stage at the time of initial diagnosis and about 30% of them develop distant metastasis [20]. With the increasing prevalence of diabetes worldwide, its pathologic roles in carcinogenesis and metastasis have attracted special attention [21, 22]. As evidenced by a meta-analysis based on individual-participant data from 97 prospective studies, the concurrence of diabetes was observed to double the risk of cancer mortality [23]. Subsequent prospective studies indicated that pre-diagnostic hyperglycemia and diabetes were significantly risk factors for cancer mortality [24–26]. Extending the findings of previous studies on the relationship between preoperative fasting blood glucose and postoperative esophageal cancer mortality, we elicited a subset of data from the ongoing Fiesta study initiated in early 2000 and over 15-year follow-up we found that the survival was much worse in ESCC patients with the elevated preoperative fasting blood glucose or diabetes than those without, and the prognostic utility was more obvious in males, the findings opposing to that of the study by Backemar et al, who in a Swedish population failed to observes an elevated risk of mortality in diabetic patients after esophageal cancer surgery [13]. A note of caution should be sounded that the prognosis of female ESCC patients was better than that of males after three-field lymphadenectomy in this study, which is most likely due to many clinical factors, such as a more favorable lifestyle and general status [27]. As exemplified by smoking status, the overall survival was significantly better in never smokers than in even smokers in male patients, whereas no significance was observed in female patients (Supplementary Figure S1). Moreover, it is widely recognized that elevated glucose consumption is a necessary component for carcinogenesis [28]. The possible mechanism underlying the relationship between glucose metabolism and cancer progression is that glucose metabolism can result in increased production of acid, which provides an evolutionary advantage to cancer cells vis-à-vis normal parenchyma into which they invade [28]. Also considering that some glycolysis-related genes were observed to be ubiquitously over-expressed in cancer cells [29], to elucidate the molecular mechanisms of glucose-triggered carcinogenesis is thereby of fundamental importance.

Several strengths distinguishing the current study merit adequate consideration. Firstly, the current cohort study involved 2396 ESCC patients who underwent three-field lymphadenectomy at the Fujian Provincial Cancer Hospital, and this sample size was so far the largest for the postoperative prognosis of esophageal cancer in medical literature. Secondly, this ongoing study has a long duration of follow-up from the year 2000 to 2015 and the minimal survival time was 5 years, which avoided potential selection biases. Thirdly, during 15 years of follow-up, our findings demonstrated for the first time that the prognosis of preoperative fasting blood glucose or diabetes for ESCC mortality was gender-independent. This finding is not surprising as esophageal cancer is more common in males than in females and divergent risk profiles have been proposed for esophageal cancer between genders [30]. Importantly, the prognostic utility of fasting blood glucose was independent of obesity in this study and was more obvious in males, especially for the early stage of ESCC patients after three-field lymphadenectomy. The clinical implications of this study underscore the urgency for the preoperative surveillance of fasting blood glucose in the evaluation of postoperative patients with early-stage ESCC in clinical practice.

Despite these strengths, several limitations of this study should be considered. The first limitation is the single-center design as all assessable patients were consecutively enrolled from the Fujian Provincial Cancer Hospital during the period between January 2000 and December 2010. The second limitation is that the findings derived in this study cannot be directly extrapolated to general populations as our patients had a 5-year survival rate of 52.15%, which was markedly higher than that of general populations (around 20%) [3, 4]. The third limitation is that although this is so far the largest prospective study for ESCC mortality, the number of patients diagnosed as EAC and esophageal neuroendocrine carcinomas is relatively small, limiting further exploratory data analyses. Future studies with larger sample sizes and longer follow-up periods specifically designed to assess the prognosis of preoperative fasting blood glucose or diabetes in patients with the two rare types of esophageal cancer are needed.

To sum up, on the basis of 2396 postoperative patients with ESCC from the FIESTA study, our findings convincingly demonstrated that the elevated preoperative fasting blood glucose can predict poor survival of ESCC patients, and this prediction was much better in males. For practical reasons, the findings of this study can help guide risk management strategies for therapeutic interventions for esophageal cancer. Pending successful validation in other independent cohorts, our findings could provide the foundation for future personalized medicine whereby ESCC patients who have elevated fasting blood glucose or diabetes with a high probability for poor survival can be identified early and treated with optimal regimens.

## MATERIALS AND METHODS

### Study patients

All study patients were consecutively selected from the Department of Thoracic Surgery, Fujian Provincial Cancer Hospital during the period between January 2000 and December 2010. The Ethical Committee of Fujian Provincial Cancer Hospital approved this study, and written informed consent was obtained from all patients.

### Eligibility criteria

Only patients who underwent three-field lymphadenectomy for esophageal cancer and were safely discharged were eligible for inclusion. The diagnosis of esophageal cancer should be confirmed by preoperative biopsies or postoperative pathologic analyses. In addition, eligible patients were Han Chinese and hospitalized for the first time for lymphadenectomy. They also had no history of any malignancies and received no preoperative or postoperative chemotherapy and radiotherapy.

### Tissue collection

From each patient, a pair of esophageal cancer tissue and matched normal esophagus tissue was sliced, and all tissues were fixed with 10% neutral formalin embedded in paraffin. Clinicopathological analyses of tissue samples were done at the Department of Pathology, Fujian Provincial Cancer Hospital.

### Follow-up assessment

Clinical outcomes were assessed every half to one year after discharge at the Out-Patient Department or through phone or post mail in case of no-show at the scheduled time. All patients were followed up from the day they entered this study until the day they died or the last time they received follow-up checking, whichever came first. As of December 2015, 147 patients were lost to follow-up and 204 patients died from causes other than esophageal cancer, leaving 2535 assessable patients in the final analysis. The median follow-up time was 44.0 months (range: 0.5 to 180 months). Over a 15-year follow-up period, 1265 of 2535 patients were recorded to be dead from esophageal cancer and there were 1270 survivors.

### Patient characteristics

All patients who had normal diets were ordered to be fasted for at least 8 hours before surgery, and venous fasting blood samples (4 mL) were drawn into the EDTA-K2 anticoagulative tubes. Fasting blood glucose was measured by an automated glucose oxidase method and diabetes mellitus was diagnosed if the fasting blood glucose ≥ 7.0 mmol/L.

At the time of enrollment, each patient was invited to fill in a self-designed questionnaire to glean socio-demographic and anthropometric information, including date of birth, age of onset for esophageal cancer, gender, body weight, body height, smoking, drinking and family cancer history. In detail, age was defined as the age at the time of surgery for esophageal cancer. Body mass index was calculated as weight (in kilograms) divided by the square of height (in meters). Smoking status was classified as never smoking and ever smoking (including formerly and currently smoking). Similarly, drinking status was classified as never drinking and ever drinking (including formerly and currently drinking). A positive family cancer history referred to one or more of affected relatives within three generations who suffered malignancies except for non-melanoma skin cancer. Diagnosis was presented if fasting blood glucose ≥ 7.0 mmol/L.

Clinicopathologic characteristics were obtained from pathological reports, including histological type (ESCC, EAC and esophageal neuroendocrine carcinomas), tumor size (in centimeters), tumor node metastasis or TNM stage (I, II, III and IV according to the 7^th^ Edition of the AJCC cancer staging manual) [31], depth of invasion (T1, T2, T3 and T4), regional lymph node metastasis or LNM (N0, N1, N2 and N3), distant metastasis (M0 and M1), tumor location (upper, middle and lower esophagus), histological differentiation (well, moderate and poor differentiation) and vascular cancer embolus (positivity and negativity).

### Statistical analysis

Data are expressed as mean ± standard deviation (s.d.) or percentage, and the differences between genders were compared by the t test or the Mann-Whitney U Test or the χ^2^ test where appropriate. Kaplan-Meier curves coupled with Log-rank tests were used to detect cumulative survival differences across patients with different characteristics. Adjusted risk estimates (hazard ratio or HR and its 95% confidence interval or 95% CI) for mortality were calculated using the multivariate Weibull proportional hazards regression analyses mainly because the ln(-ln(S(t))) is a linear function of ln(t) (here, t is time variable, and S(t) is survival function). Survival tree structure was depicted by the STREE software (http://c2s2.yale.edu/software/stree/) to classify patients into subgroups with distinct median survival time (MST). All statistical tests were two-sided and a probability of less than 0.05 was considered to be statistically significant. All statistical analyses were run with the STATA software for Windows (StataCorp, TX, USA, version 13.0).

## SUPPLEMENTARY FIGURES


