# Supplementary material for: The elevated preoperative fasting blood glucose predicts a poor prognosis in patients with esophageal squamous cell carcinoma: The Fujian prospective investigation of cancer (FIESTA) study
Source: Oncotarget. 2016 Aug 12;7(40):65247–56. doi: 10.18632/oncotarget.11247 (PMC5323152; doi:10.18632/oncotarget.11247)
Supplement: Supplementary file 1 [file oncotarget-07-65247-s001.pdf]

The elevated preoperative fasting blood glucose predicts a poor prognosis in patients with esophageal squamous cell carcinoma: The Fujian prospective investigation of cancer (FIESTA) study

SUPPLEMENTARY FIGURES

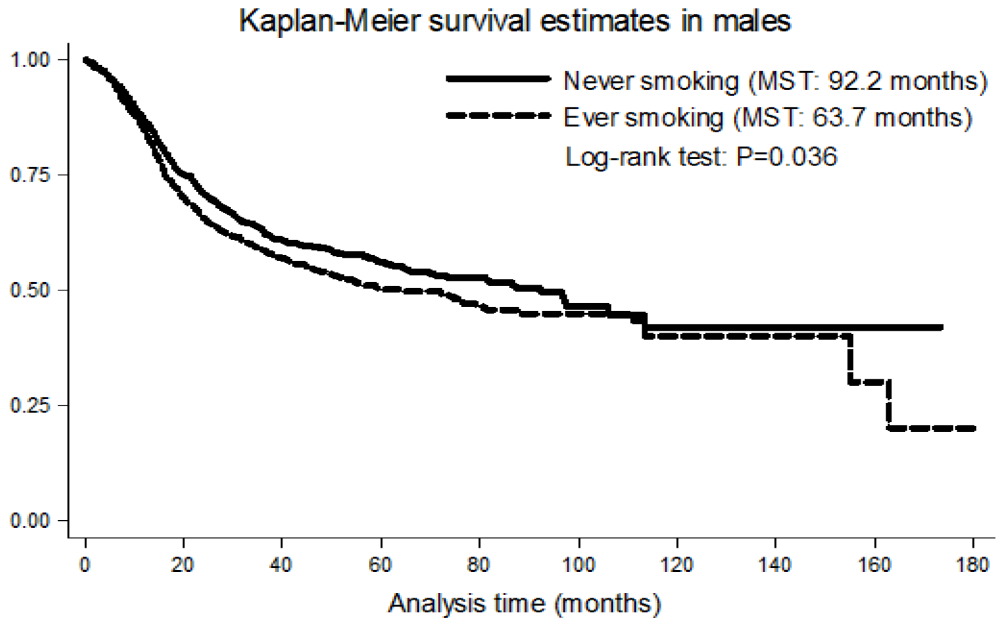

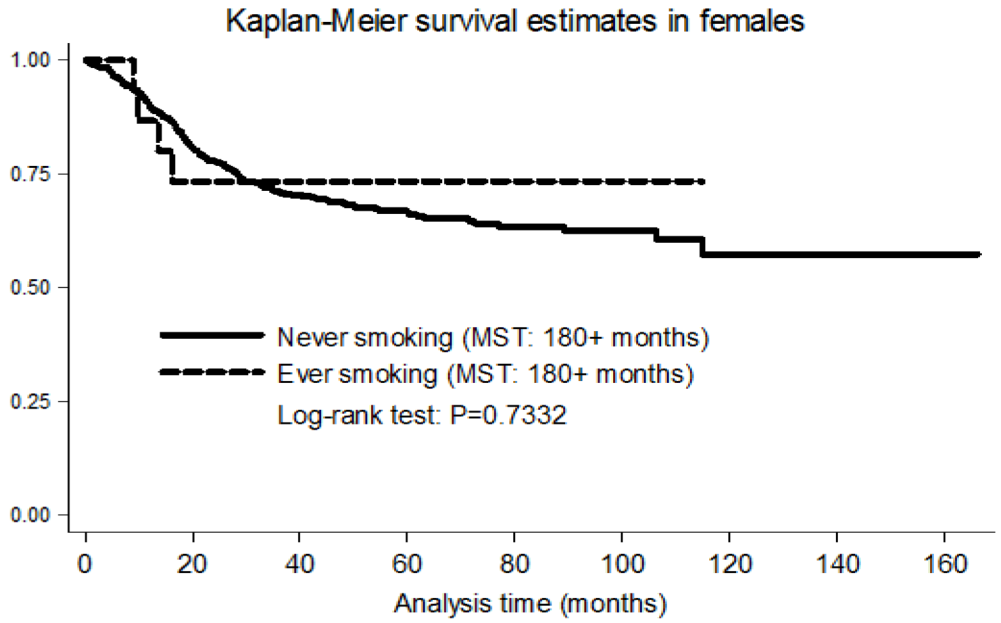

Supplementary Figure S1: The Kaplan-Meier survival curves by smoking in both genders.
Abbreviations: MST, median survival time. The vertical axis represents the cumulative survival rate.

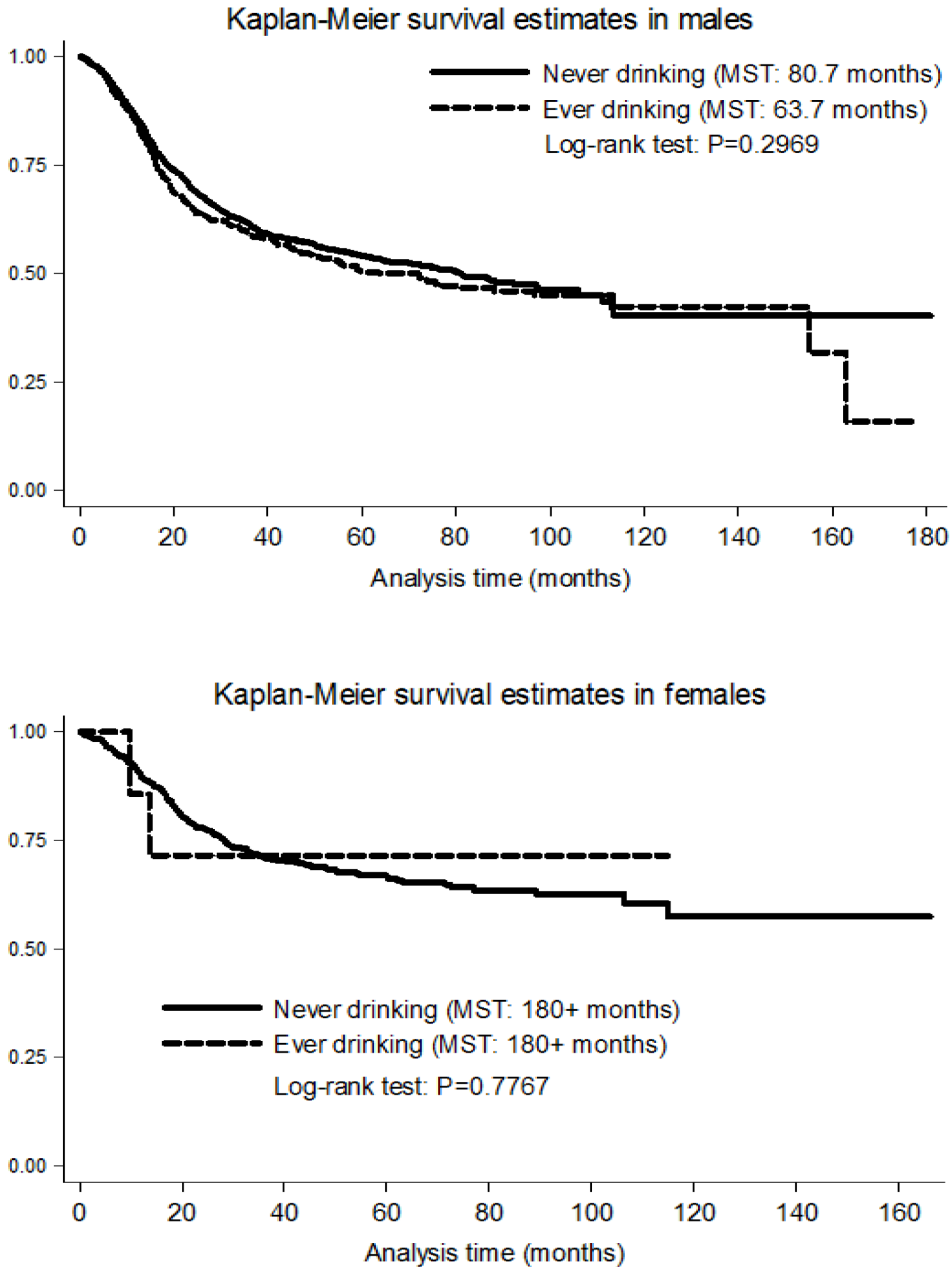

Supplementary Figure S2: The Kaplan-Meier survival curves by drinking in both genders.
Abbreviations: MST, median survival time. The vertical axis represents the cumulative survival rate.

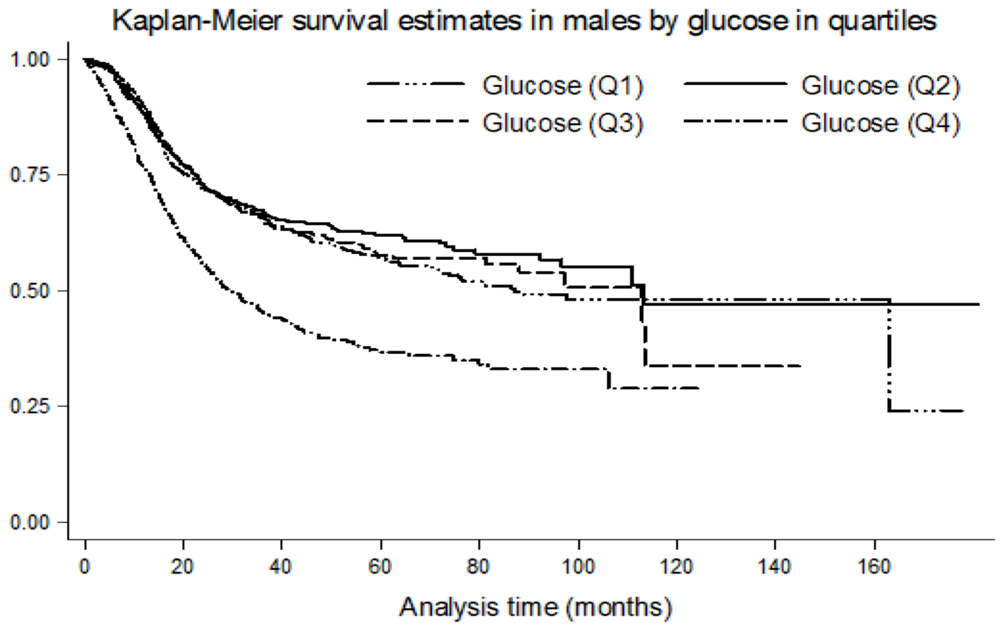

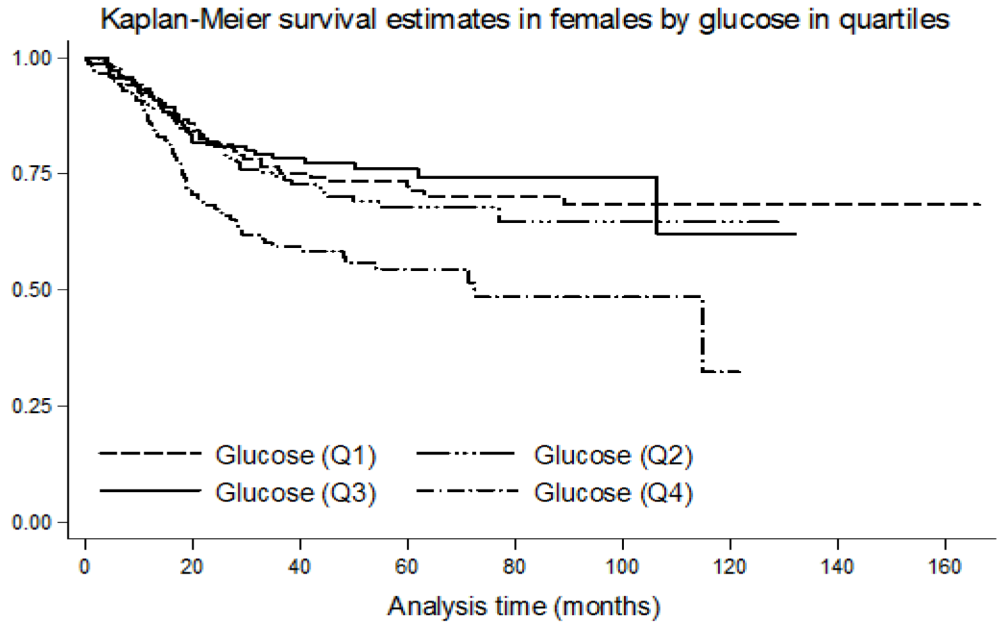

Supplementary Figure S3: The Kaplan-Meier survival curves by fasting blood glucose in quartiles in both genders. *Abbreviations*: MST, median survival time. The vertical axis represents the cumulative survival rate.
